# Navafenterol (AZD8871) in patients with mild asthma: a randomised placebo-controlled phase I study evaluating the safety, tolerability, pharmacokinetics, and pharmacodynamics of single ascending doses of this novel inhaled long-acting dual-pharmacology bronchodilator

**DOI:** 10.1186/s12931-020-01470-5

**Published:** 2020-09-09

**Authors:** Eulalia Jimenez, Carol Astbury, Muna Albayaty, Ulrika Wählby-Hamrén, Beatriz Seoane, Cristina Villarroel, Helena Pujol, Maria Jesus Bermejo, Ajay Aggarwal, Ioannis Psallidas

**Affiliations:** 1 Clinical Pharmacology and Quantitative Pharmacology, Clinical Pharmacology & Safety Sciences, R&D, AstraZeneca, 08020 Barcelona, Spain; 2 Research and Early Development, Respiratory, Inflammation and Autoimmune, BioPharmaceuticals R&D, AstraZeneca, Barcelona, Spain; 3 Early Phase Clinical Unit, PAREXEL International GmbH, Harrow, UK; 4 Clinical Pharmacology and Quantitative Pharmacology, Clinical Pharmacology & Safety Sciences, R&D, AstraZeneca, Gothenburg, Sweden; 5 Biometrics and Information Sciences, Late-Stage Development, BioPharmaceuticals R&D, AstraZeneca, Barcelona, Spain; 6 Late-Stage Development, BioPharmaceuticals R&D, AstraZeneca, Barcelona, Spain; 7 Patient Safety, Chief Medical Office, R&D, AstraZeneca, Barcelona, Spain; 8 Research and Early Development, Respiratory, Inflammation and Autoimmune, BioPharmaceuticals R&D, AstraZeneca, Gothenburg, Sweden

**Keywords:** Bronchodilator, Asthma, MABA, dual-pharmacology muscarinic receptor antagonist β_2_-adrenoceptor agonist, Safety, Pharmacokinetics

## Abstract

**Background:**

Navafenterol (AZD8871) is an inhaled long-acting dual-pharmacology muscarinic antagonist/β_2_-adrenoceptor agonist (MABA) in development for the treatment of obstructive airways diseases. The safety, tolerability, pharmacodynamics, and pharmacokinetics of navafenterol were investigated in patients with mild asthma.

**Methods:**

This was a randomised, single-blind, placebo-controlled, single-ascending-dose study. Patients were randomly assigned to one of two cohorts which evaluated escalating doses of navafenterol (50–2100 μg) in an alternating manner over three treatment periods. The primary pharmacodynamic endpoint was the change from pre-dose baseline in trough forced expiratory volume in 1 s (FEV_1_) for each treatment period.

**Results:**

Sixteen patients were randomised; 15 completed treatment. Data from all 16 patients were analysed. The maximum tolerated dose was not identified, and all doses of navafenterol were well tolerated. The most frequently reported treatment-emergent adverse events (TEAEs) were headache (*n* = 10, 62.5%) and nasopharyngitis (*n* = 7, 43.8%). No TEAEs were serious, fatal, or led to discontinuation, and no dose dependency was identified. Navafenterol demonstrated a dose-ordered bronchodilatory response with a rapid onset of action (within 5 min post-dose). Doses ≥200 μg resulted in improvements in trough FEV_1_ (mean change from baseline range 0.186–0.463 L) with sustained bronchodilation for 24–36 h. Plasma concentrations increased in a dose-proportional manner, peaking ~ 1 h post-dose, with a derived terminal elimination half-life of 15.96–23.10 h.

**Conclusions:**

In this study navafenterol was generally well tolerated with a rapid onset of action which was sustained over 36 h.

**Trial registration:**

ClinicalTrials.gov; No.: NCT02573155

## Background

Inhaled bronchodilators are used widely in the symptomatic management of asthma [1]. The long-acting muscarinic antagonist (LAMA) bronchodilator tiotropium is recommended as an add-on to dual therapy with an inhaled corticosteroid (ICS) and a long-acting β_2_ receptor agonist (LABA) bronchodilator for the treatment of patients with severe asthma that is not well controlled with ICS/LABA therapy [1]. Modest improvements in lung function [2–4] and a decreased risk of severe exacerbations requiring oral corticosteroids have been reported with this combination of therapies [2, 3].

Compared with LAMA/LABA combinations, a single molecule with dual muscarinic antagonist and β_2_ agonist (MABA) properties may be more readily co-formulated with ICS and/or novel anti-inflammatory agents [5, 6], potentially simplifying dosing regimens and improving patient compliance [5, 7–9]. Additionally, the use of a single bifunctional molecule allows the delivery of a fixed drug ratio into every region of the lung and a uniform ratio of activities at the cellular level, and offers the potential for additive or synergistic bronchodilation vs bronchodilators with a single mechanism of action [5, 10, 11].

Navafenterol (AZD8871) is a novel MABA currently being developed as an inhaled long-acting bronchodilator (e-Fig. 1). Through its dual pharmacological activity, it is anticipated that navafenterol would offer greater efficacy than single-mechanism LABAs or LAMAs, and similar or potentially greater efficacy than free- or fixed-dose combination therapies, with an equivalent or superior safety and tolerability profile. This first-in-human phase I study (NCT02573155) was conducted in two parts: a single-ascending-dose study in male patients with mild asthma (Part 1) and a 5-way cross-over, single-dose study in patients with moderate to severe chronic obstructive pulmonary disease (COPD; Part 2). In Part 2, single doses of navafenterol at 400 and 1800 μg were well tolerated and produced rapid and sustained bronchodilation over 36 h [12]. Here, we report data from Part 1 of the study, which was designed to evaluate the safety, tolerability, pharmacokinetics (PK), and pharmacodynamics (PD) of single doses of navafenterol in patients with mild asthma.

## Methods

### Study design and treatment

This was a randomised, single-blind, placebo-controlled study conducted at a single site (PAREXEL Early Phase Clinical Unit, Northwick Park Hospital, Harrow, UK). The primary objectives were to assess the safety, tolerability, and PD of single doses of navafenterol, and the secondary objective was to assess the PK of navafenterol and its metabolite after single doses of navafenterol. The study protocol and consent form were reviewed and approved by an Independent Ethics Committee (NRES Committee – Cambridgeshire and Hertfordshire, Health Research Authority, Nottingham, UK; Reference No 15/EE/0329) and the study was performed in accordance with the Declaration of Helsinki, the International Conference on Harmonisation/Good Clinical Practice and the AstraZeneca bioethics policy. All patients provided written informed consent before taking part in the study.

**Fig. 1:**
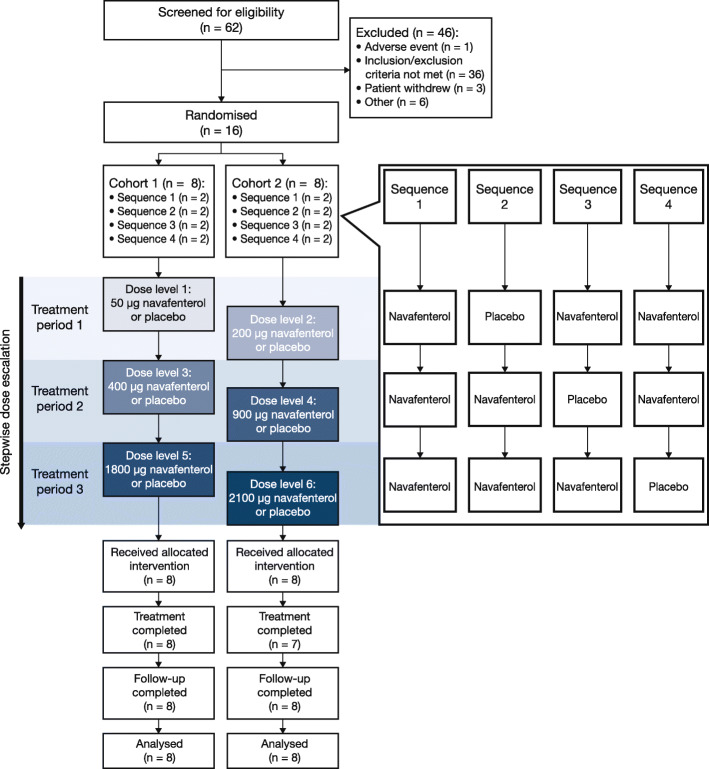
Patient disposition and flow

Following a screening evaluation and run-in period of 14 to 28 days, patients were withdrawn from their usual non-ICS asthma therapies. Patients receiving ICS monotherapy were maintained on their usual ICS therapy, and patients receiving fixed-dose combinations of ICS/LABA were permitted to switch to the same ICS but in a monotherapy formulation. Patients were randomly assigned to one of two cohorts: each cohort received one of six navafenterol dose levels per treatment period (Fig. 1). Doses were sequentially escalated over the three treatment periods, separated by a minimum washout period of 14 days. During each treatment period, patients received either navafenterol or placebo (both delivered using a variant of the Genuair™/Pressair®^1^ dry-powder inhaler, adapted internally to deliver a single dose of inhalation powder), at the same time each morning. Salbutamol was permitted as reliever medication when required for the duration of the study. A follow-up visit was performed 7 (±2) days after the last treatment period or after discontinuation, and patients were contacted by telephone 14 (±2) days after the last treatment period to record any adverse events (AEs; defined as undesirable medical conditions which developed or deteriorated at any time during the study, irrespective of causality). Treatment-emergent AEs (TEAEs) were defined as AEs that developed (or increased in severity) after the first administration of the study drug and within 14 days of the last administration of the study drug.

### Participants

Eligible patients were males aged 18–70 years with a body mass index of 18–32 kg/m^2^ and a clinical diagnosis of asthma (according to the Global Initiative for Asthma guidelines) for at least 6 months prior to screening. For inclusion, patients were required to: be non-smokers with a smoking history of ≤10 pack-years; have a forced expiratory volume in 1 s (FEV_1_) value of ≥70% of the predicted normal at screening; and have FEV_1_ reversibility of ≥12% and an absolute increase of ≥200 mL vs baseline within 30 min after inhalation of 400 μg of salbutamol. Patients were otherwise healthy, as determined by medical history, physical examination, and 12-lead digital electrocardiogram (ECG) findings. Key exclusion criteria included a current diagnosis of COPD or other clinically significant unstable disease, and increased maintenance treatments for asthma or an asthma exacerbation requiring hospitalisation within the 6 weeks prior to screening or randomisation.

### Assessments

#### Safety and tolerability

AEs, coded using the Medical Dictionary for Regulatory Activities version 18.1, were collected at each visit from consent until the telephone follow-up. Other safety assessments included physical examinations, vital signs (blood pressure and body temperature), clinical laboratory tests (chemistry; haematology; urinalysis; coagulation; and blood potassium and glucose measured using i-STAT), 12-lead digital ECG, and telemetry. For the timing of these assessments and the safety stopping criteria see e-Appendix 1 and e-Table 1.

#### Pharmacodynamics

The primary PD endpoint was the change from baseline (measured prior to the administration of study drug on day 1) in trough FEV_1_ (mean of two values measured at 23 and 24 h after the administration of study drug on day 1) for each treatment period. Key secondary endpoints included: the change from baseline in trough forced vital capacity, normalised FEV_1_ area under the curve from 0 to 6 (AUC_0–6_), 0–12 (AUC_0–12_), 12–24 (AUC_12–24_), and 0–24 (AUC_0–24_) h post-dose, and FEV_1_ at each scheduled timepoint on day 1 and day 2; and peak FEV_1_ on day 1. For the timing of these assessments see e-Appendix 1.

#### Pharmacokinetics

The PK of navafenterol and its metabolite LAS191861 were assessed in plasma and urine. The plasma PK parameters assessed included the maximum plasma concentration (C_max_), time to reach C_max_ (t_max_), concentration-time AUC_0–24_, area under the concentration-time curve from time zero extrapolated to infinity (AUC_0–∞_), and terminal elimination half-life (t_½λz_). Renal clearance and the fraction of the dose excreted unchanged into urine from 0 to 36 h (fe_0-36h_) were also assessed. For details of the sampling schedule and parameter assessments see e-Appendix 1.

### Statistical analysis

Due to the exploratory nature of this trial, there was no formal sample size calculation. It was considered that a sample size of 16 patients would be sufficient to meet the objectives of the study. SAS version 9.2 or later (SAS Institute, Inc., Cary, NC) was used for all analyses.

Safety outcomes were analysed descriptively in the safety population (all randomised patients who received at least one dose of study medication). Analyses of PD were performed on the per protocol population; defined as all randomised patients who met the inclusion/exclusion criteria, completed at least one treatment period, and had no major protocol deviations. PK parameters were analysed descriptively in the PK population (all randomised patients with evaluable PK parameters who received at least one dose of study medication in any treatment period). Additional information regarding the statistical analyses can be found in e-Appendix 1.

## Results

### Participants

The first patient was enrolled on October 30, 2015 and the last patient completed the study on March 24, 2016. Of the 62 patients screened for eligibility, 16 were randomised to treatment (Fig. 1). Fifteen patients (93.8%) completed all scheduled treatments and the study. One patient was withdrawn before receiving navafenterol 2100 μg during treatment period 3 after receiving treatment with amoxicillin and ibuprofen for pharyngitis. However, all randomised patients completed follow-up and were included in the safety, per protocol and PK populations. All patients showed reversibility at screening (Table 1).

**Table 1:** Patient Demographics and Baseline Characteristics (Safety Population)

Baseline characteristic	Total population (*N* = 16)
Age, years	39.0 (11.8)
Male, n (%)	16 (100)
Race, n (%)	
Asian	1 (6.3)
Black or African American	1 (6.3)
White	14 (87.5)
Weight, kg	82.5 (15.6)
Height, cm	174.9 (7.9)
Body mass index, kg/m^2^	26.8 (3.5)
Smoking status, n (%)	
Former	1 (6.3)
Never	15 (93.8)
Asthma severity per GINA 2006 stages, n (%)	
Category I^a^	8 (50.0)
Category II^b^	8 (50.0)
Duration of asthma, years	25.9 (11.5)
Prebronchodilator % predicted FEV_1_	80.8 (9.3)
Bronchial reversibility, %	20.2 (7.9)
Prior medication^c^ for asthma, n (%)	1 (6.3)

*FEV*_*1*_ forced expiratory volume in 1 s, *GINA* Global Initiative for Asthma

Data are mean (standard deviation) unless otherwise specified

^a^FEV_1_ ≥ 80% of predicted value (mild persistent)

^b^FEV_1_ ≥ 60% of predicted value and < 80% of predicted value

^c^Within 15 days prior to providing informed consent until the first administration of study medication

### Safety and tolerability

After reviewing emerging data from each preceding dose level, as per protocol, the planned dose escalations for dose levels 2–6 (100 μg, 300 μg, 600 μg, 1200 μg, and 1800 μg) were increased to 200 μg, 400 μg, 900 μg, 1800 μg, and 2100 μg. The maximum tolerated dose was not identified in this study. Safety stopping criteria were not met and the predefined human exposure limit (determined from non-clinical toxicology investigations) was not reached following dose escalation to 2100 μg.

Overall, 14 (87.5%) patients reported ≥1 TEAE and 4 (25.0%) patients reported treatment-related TEAEs. The most frequently reported TEAEs were headache (*n* = 10, 62.5%) and nasopharyngitis (*n* = 7, 43.8%; Table 2), and treatment-related TEAEs included headache (*n* = 3, 18.8%), dizziness (n = 1, 6.3%), and cough (n = 1; 6.3%). The incidence of TEAEs and treatment-related TEAEs was similar across treatment groups and dose levels, and all TEAEs were considered to be of mild or moderate intensity; none were severe, serious, fatal, or led to discontinuation. Furthermore, no TEAEs were related to laboratory findings.

**Table 2:** Summary of TEAEs by Treatment and Dose (Safety Population)

	Placebo	navafenterol 50 μg	navafenterol 200 μg	navafenterol 400 μg	navafenterol 900 μg	navafenterol 1800 μg	navafenterol 2100 μg	Overall
N	12	6	6	6	6	6	5	16
Any TEAE, n (%)	7 (58.3)	5 (83.3)	5 (83.3)	3 (50.0)	3 (50.0)	3 (50.0)	2 (40.0)	14 (87.5)
Most common TEAEs_,_ n (%)^a^								
Headache	3 (25.0)	3 (50.0)	1 (16.7)	2 (33.3)	2 (33.3)	1 (16.7)	0	10 (62.5)
Nasopharyngitis	1 (8.3)	3 (50.0)	1 (16.7)	0	0	0	2 (40.0)	7 (43.8)
Chest discomfort	1 (8.3)	1 (16.7)	1 (16.7)	0	0	0	0	3 (18.8)
Skin irritation	0	1 (16.7)	1 (16.7)	0	0	1 (16.7)	0	3 (18.8)
Wheezing	1 (8.3)	1 (16.7)	1 (16.7)	0	0	0	0	3 (18.8)
Cough	0	1 (16.7)	0	0	1 (16.7)	0	0	2 (12.5)
Dizziness	1 (8.3)	0	1 (16.7)	0	0	0	0	2 (12.5)
Feeling hot	0	1 (16.7)	0	0	0	0	1 (20.0)	2 (12.5)
Nausea	0	1 (16.7)	0	0	1 (16.7)	0	0	2 (12.5)

*TEAE* treatment-emergent adverse event

Adverse events were coded using Medical Dictionary for Regulatory Activities version 18.1

^a^Most common TEAEs reported by ≥2 patients overall

**Fig. 2:**
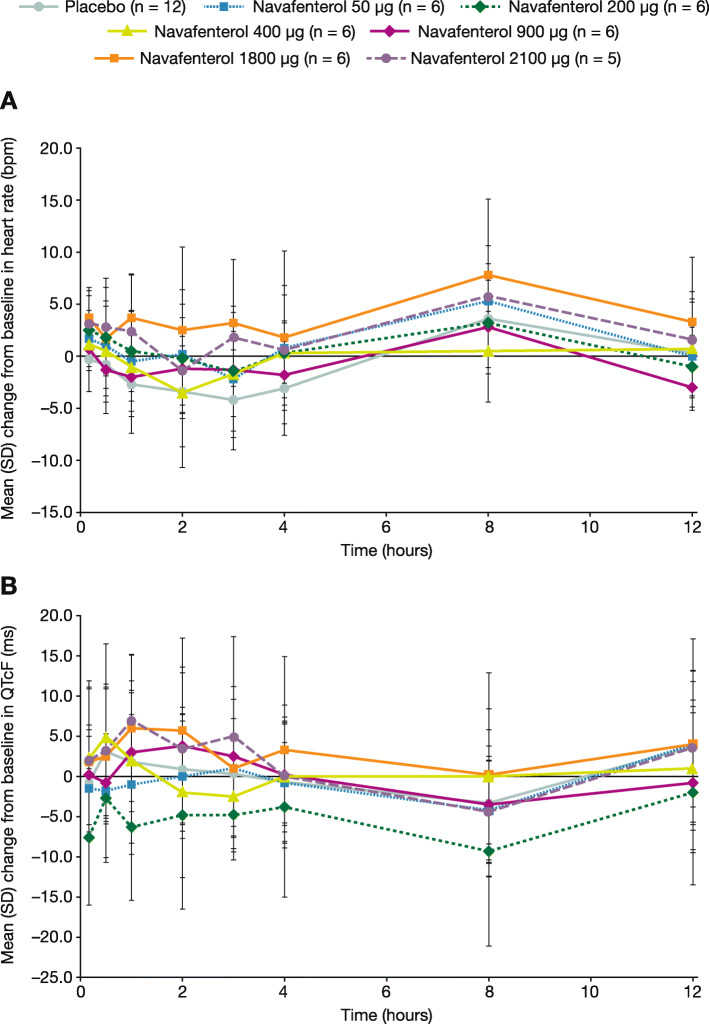
Mean (SD) change from baseline in (**a**) heart rate and (**b**) QTcF at each timepoint over 12 h post-dose (safety population). bpm = beats per minute; QTcF = QT interval corrected for heart rate using the Fridericia formula; SD = standard deviation

There were no clinically significant changes in clinical laboratory parameters, vital signs, or ECGs. Small increases in heart rate and changes from baseline in mean QT interval corrected for heart rate using the Fridericia formula (QTcF) were observed, and were comparable across all treatment groups and dose levels (Fig. 2). However, mean QTcF values remained within the reference ranges for all doses at all timepoints. Small changes in i-STAT mean glucose and potassium values were observed for patients receiving placebo or navafenterol, but there was no observed pattern for the changes (e-Fig. 2).

### Pharmacodynamics

**Fig. 3:**
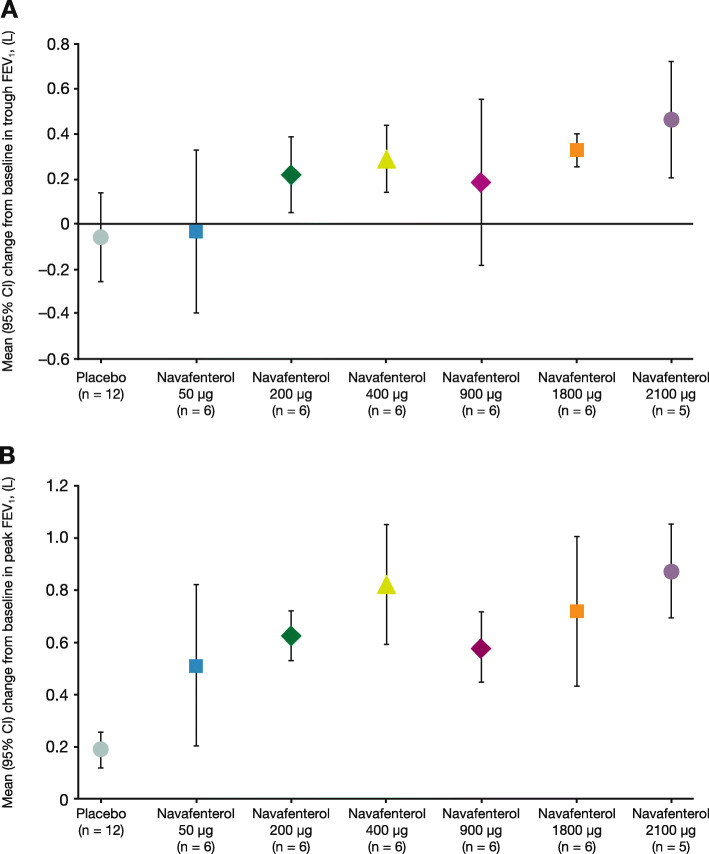
Mean (95% CI) change from baseline in **a**) trough and **b**) peak FEV_1_ (per protocol population). CI = confidence interval; FEV_1_ = forced expiratory volume in 1 s

The mean change from baseline in trough FEV_1_ (primary PD endpoint) increased with higher doses of navafenterol, demonstrating a dose-ordered bronchodilatory response (Fig. 3). Improvements, ranging from 0.186 to 0.463 L vs baseline, were observed with navafenterol doses ≥200 μg. Based on the results of an exploratory analysis of covariance, the improvements in the least squares mean change from baseline in trough FEV_1_ were statistically superior for navafenterol vs placebo at doses of 400 μg (0.301 L; 95% confidence interval [CI] 0.195, 0.406), 900 μg (0.284 L; 95% CI 0.176, 0.391), 1800 μg (0.389 L; 95% CI 0.134, 0.643), and 2100 μg (0.414 L; 95% CI 0.154, 0.673).

Administration of navafenterol resulted in a rapid onset of action, with all doses numerically increasing FEV_1_ vs baseline at both 5 and 15 min post-dose (e-Fig. 3). Bronchodilation was also sustained, with all doses ≥200 μg numerically increasing FEV_1_ vs baseline from 24 to 36 h post-dose. The change from baseline in peak FEV_1_ was increased by all doses of navafenterol, ranging from 0.513 to 0.870 L (Fig. 3 and e-Table 2). Other secondary PD endpoints, including the change from baseline in trough forced vital capacity and post-dose normalised FEV_1_ AUC_0–6_, AUC_0–12_, AUC_12–24_, and AUC_0–24_ are reported in e-Table 2.

### Pharmacokinetics

**Fig. 4:**
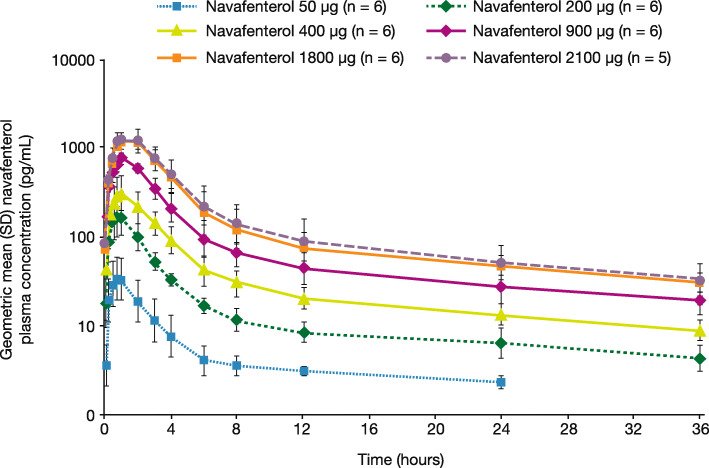
Geometric mean (SD) navafenterol plasma concentration at each timepoint over 36 h post-dose (pharmacokinetics population). Data are shown on a semi-logarithmic scale. For error bars, the geometric mean SD is displayed as exponential (arithmetic mean of the natural log-transformed variable ± arithmetic SD of the natural log-transformed variable). SD = standard deviation

Following single inhalation, navafenterol was rapidly absorbed (median t_max_ values of 0.86–1.49 h; e-Table 3). C_max_ and AUC_0–∞_ increased dose proportionally across the dose range, according to the statistical analysis. After reaching C_max_, plasma concentrations declined in an apparent biphasic manner (Fig. 4), with mean t_½λz_ ranging from 15.96 to 23.10 h with navafenterol doses ≥200 μg. Across all doses fe_0-36h_ was very low, with < 0.4% excreted unchanged in urine. Renal clearance was consistently low, with mean values of 0.7–1.1 L/h.

The metabolite LAS191861 was rapidly formed following inhalation of navafenterol (median t_max_ 0.99–3.00 h). Mean metabolite to parent ratios based on AUC_0–∞_ ranged from approximately 0.12 to 0.24, independent of dose.

## Discussion

In this study of patients with mild asthma, navafenterol had a rapid onset of action, with improvements in bronchodilation vs baseline observed at 5 min post-dose. At doses of 400–2100 μg navafenterol demonstrated improvements from baseline in trough FEV_1_ that were statistically superior to placebo. A dose-ordered increase was observed for the changes from baseline in trough FEV_1_. After peak FEV_1_ was reached, the bronchodilatory effect gradually declined but was still sustained until the end of the observation period at 36 h post-dose.

The PK profile of navafenterol in plasma showed rapid absorption and dose-proportional exposure for navafenterol. Though mean t_½λz_ estimates were generally consistent across doses of navafenterol, these values were generally calculated over a period of less than three times the resultant half-life for all dose levels, which may impact the robustness of the values. A very low fraction of the dose was excreted unchanged in the urine, and low renal clearance values were suggestive of passive filtration mechanisms.

Navafenterol at doses of 50–2100 μg was generally well tolerated. No stopping criteria were reached during the study and no safety concerns were identified. Dosing remained below pre-identified maximum human exposure limits and the maximum tolerated dose was not reached.

No TEAEs were fatal, serious, severe, or led to discontinuation, and the proportion of patients reporting TEAEs was similar across treatment groups and dose levels. No TEAEs were related to laboratory findings, and no clinically significant findings were reported for clinical laboratory parameters. No prominent adrenergic symptoms were observed, and there was no indication of any cardiovascular effects, clinically relevant differences in blood pressure, or treatment-related hyperglycaemia or hypokalaemia.

Taken together, the safety and PD findings of this first-in-human study of navafenterol support the once-daily dosing of navafenterol as an inhaled bronchodilator and warrant further clinical investigation. However, the conclusions that can be drawn from this study are limited by the small sample size and the choice of treatment population. Though this was considered to be the most sensitive population to demonstrate early signs of potential efficacy without compromising patient safety, it is not representative of the intended target population. Currently, navafenterol is being developed for use in COPD. Further studies would be required to confirm the efficacy of navafenterol in patients with COPD and in patients with severe asthma in combination with an ICS and/or another anti-inflammatory agent. With its rapid onset of action and good safety profile, it is possible that navafenterol could eventually be used as an add-on reliever medication, on a similar symptom-driven, as-needed (prn) basis to the LABA formoterol, for patients with obstructive airways disease [13]. Currently, short-acting β_2_-agonists (SABA), short-acting muscarinic antagonists (SAMA), formoterol and ICS/formoterol combinations are licensed for this form of use in patients with asthma [14].

## Conclusions

In the first administration of navafenterol in humans, no safety concerns were raised and navafenterol 50–2100 μg was generally well tolerated, with significant and rapid-onset bronchodilation (within 5 min post-dose), which was sustained over 36 h. Whilst these findings are limited by the size of the study, they support further investigations of navafenterol as a dual-action inhaled bronchodilator.

## Supplementary information

**Additional file 1 e-Appendix 1**. Methods. **E-Table 1**. Safety Assessments. **E-Table 2**. Secondary Pharmacodynamic Endpoints. **E-Table 3**. PK Parameters for navafenterol. **e-Figure 1**. The chemical structure of navafenterol. **e-Figure 2**. Mean (SD) change from baseline in (A) serum glucose and (B) serum potassium over 24 h post-dose. **e-Figure 3**. Mean (95% CI) change from baseline in FEV_1_ at each timepoint over 36 h post-dose.

## Data Availability

Data underlying the findings described in this manuscript may be obtained in accordance with AstraZeneca’s data sharing policy described at https://astrazenecagrouptrials.pharmacm.com/ST/Submission/Disclosure.

## Abbreviations

AE: Adverse event
AUC0–∞: Area under the concentration-time curve from time zero extrapolated to infinity
AUC0–6: Area under the curve from 0 to 6 h
AUC0–12: Area under the curve from 0 to 12 h
AUC0–24: Area under the curve from 0 to 24 h
AUC12–24: Area under the curve from 12 to 24 h
CI: Confidence interval
Cmax: Maximum concentration
COPD: Chronic obstructive pulmonary disease
ECG: Electrocardiogram
fe0-36 h: Fraction of the dose excreted unchanged into urine from 0 to 36 h
FEV1: Forced expiratory volume in 1 s
ICS: Inhaled corticosteroid
LABA: Long-acting β2 adrenoceptor agonist
LAMA: Long-acting muscarinic receptor antagonist
MABA: Muscarinic receptor antagonist and β2 adrenoceptor agonist
PD: Pharmacodynamics
PK: Pharmacokinetic
prn: pro re nata (as needed)
QTcF: QT interval corrected for heart rate using the Fridericia formula
t½λz: Terminal half-life
TEAE: treatment-emergent adverse event
tmax: Time to reach maximum concentration

## Footnotes

1 Registered trademarks of the AstraZeneca group of companies; for use within the USA as Pressair® and Genuair™ within all other licensed territories.
