# Supplementary material for: Navafenterol (AZD8871) in patients with mild asthma: a randomised placebo-controlled phase I study evaluating the safety, tolerability, pharmacokinetics, and pharmacodynamics of single ascending doses of this novel inhaled long-acting dual-pharmacology bronchodilator
Source: Respir Res. 2020 Sep 9;21(Suppl 1):211. doi: 10.1186/s12931-020-01470-5 (PMC7487994; doi:10.1186/s12931-020-01470-5)

Online Supplement

e-Appendix 1. Methods

Study Design and Treatment

Dosing was alternated between two cohorts in order to evaluate six navafenterol (AZD8871) dose levels in an escalating and stepwise manner over approximately 10–15 weeks. This resulted in a three-treatment, three-period cross-over for each individual patient.

Within each cohort, patients were randomly assigned to one of four treatment sequences (1:1:1:1), and received either navafenterol or placebo (3:1) during each of the three treatment periods (Fig 1).

The starting dose for the study (dose level 1) was 50 µg, and five further doses were planned (100 µg, 300 µg, 600 µg, 1200 µg, and 1800 µg). The doses for all subsequent dose levels were determined after reviewing the safety, tolerability, 24-hour pharmacokinetic (PK), and pharmacodynamic (PD) data from the previous dose level. An escalation of up to 4-fold the previous dose was considered for the first three dose escalation steps (dose levels 1 to 4) and up to 2-fold for the remaining two escalation steps (dose levels 4 to 6). A larger step at the lower doses was justified as the initial doses were expected to be below the therapeutic range.

The manufactured doses of navafenterol were 50 µg, 100 µg, 300 µg, and 600 µg. Patients were required to inhale from 1 to 4 inhalers consecutively in order to receive the determined doses: 50 µg (1 × 50 µg), 200 µg (2 × 100 µg), 400 µg (100 µg, 300 µg), 900 µg (300 µg, 600 µg), 1800 µg (3 × 600 µg), and 2100 µg (3 × 600 µg, 300 µg); or matching placebo (1–4 × 0 µg).

Safety Stopping Criteria

Administration of study medication to an individual patient was stopped if the patient experienced any of the following:

- A PR interval > 250 ms at any of the electrocardiograms (ECGs) performed during the study, confirmed (persistent for > 5 min) on repeated 12-lead ECGs
- A serious adverse event (AE) considered by the safety review committee to be possibly related to study medication
- A clinically significant AE considered by the safety review committee to be possibly related to study medication, and in the opinion of the safety review committee warranting discontinuation for the patient's wellbeing.

Further dose escalation was stopped, with the possibility of early termination of Part 1 of the study, if, after the evaluation of a specific dose level, any of the following scenarios (with a reasonable possibility of a causal relationship with study medication, and where changes refer to pre-dose values from the same period) occurred:

- One or more patients who received navafenterol:
  - experienced a drug related serious AE
  - fulfilled Hy's law (defined as an increase in aspartate aminotransferase [AST] or alanine aminotransferase [ALT] ≥ 3× the upper limit of normal [ULN] and total bilirubin ≥ 2× ULN, where no other reason can be found to explain the combination of increases, eg elevated serum alkaline phosphatase [ALP] indicating cholestasis, viral hepatitis, or another drug; the elevations do not have to be at the same time or within a specified time frame)

  - had a documented tachyarrhythmia of concern, as reviewed by the safety review committee.
- Two or more patients who received navafenterol:
  - experienced paradoxical bronchospasm (had a fall in their FEV_1 ≥30% compared with the pre-dose value, associated with symptoms of dyspnoea, cough, wheezing, or chest tightness) < 2 h after administration of study medication
  - had documented symptoms of concern in the lower airways (ie dyspnoea, wheeze, chest pain, or cough which were not explained by other causes such as the common cold) < 24 h after administration of study medication, as reviewed by the safety review committee
  - had a QTc prolongation (QT interval corrected for heart rate using the Fridericia formula [QTcF] > 500 ms) or an increase of QTcF > 60 ms above baseline on the 12-lead ECG, confirmed (persistent for > 5 min) on repeated 12-lead ECGs
  - had a PR interval > 250 ms, confirmed (persistent for > 5 min) on repeated 12-lead ECGs
  - had tachycardia (resting supine heart rate > 125 beats per minute [bpm]) persisting for ≥ 10 min
  - had symptomatic bradycardia (resting supine heart rate < 45 bpm) or asymptomatic bradycardia (resting supine heart rate < 30 bpm) while awake, persisting for ≥ 10 min
  - developed hypotension (asymptomatic fall in systolic blood pressure [SBP] > 20 to < 70 mmHg persisting for at least 10 min, or symptomatic fall in resting supine SBP > 20 to < 90 mmHg)
  - developed hypertension (increase in resting supine SBP > 40 mmHg from pre-dose and to > 180 mmHg, persisting for ≤10 min on repeated assessment)

- had > 3× ULN of either ALT or AST, or > 2× ULN for bilirubin, or > 2 × ULN for ALP
- developed persistent (≥ 30 min) decreased potassium concentration (< 2.8 mmol/L; determined through i-STAT)
- had other clinically significant changes in laboratory values or other safety and tolerability parameters (eg AEs, ECG variables, or vital signs such as blood pressure) or spirometry making the continuation of study medication unjustified, as judged by the investigator
- had renal toxicity (confirmed serum creatinine increase to > 1.5 × ULN)
- had hematologic toxicity (≥ 1 confirmed count of leukocytes < 2.0 × 10^9/L, neutrophils < 1.0 × 10^9/L, or platelets < 75 × 10^9/L).

- Mean concentration-time area under the curve from 0 to 24 h (AUC_{0-24}) and mean maximum plasma concentration (C_{max}) exceeded exposure limits (7083 pg.h/mL and 1840 pg/mL, respectively).

Safety Assessments

AEs were collected at each visit from consent until the telephone follow-up (14 ± 2 days after the last dose). For the timing of other safety assessments, see e-Table 1.

Pharmacodynamic Assessments

FEV_1 and forced vital capacity were determined at screening, baseline (15 and 45 min pre-dose), 5, 15, 30, and 45 min, and 1, 2, 3, 4, 6, 8, 12, 14, 23, 24, and 36 h after the administration of study medication on day 1 of each treatment period, and at follow up. At randomisation, FEV_1 was required to be within 20% of the FEV_1 recorded at screening.

Pharmacokinetic Assessments

Blood samples were collected pre-dose and at 5, 15, 30, and 45 min, and 1, 2, 3, 4, 6, 8, 12, 24, and 36 h after the administration of study medication on day 1 of each treatment period. Samples for urine analysis were collected at baseline and at regular intervals (0–4, 4–8, 8–12, 12–24, and 24–36 h) after the administration of study medication on day 1 of each treatment period.

PK parameters were derived using non-compartmental methods with Phoenix® WinNonlin® version 6.2 (Certara USA, Inc., Princeton, NJ, USA). The concentrations of both navafenterol and LAS191861 in plasma were assessed using a single validated bioanalytical assay based on liquid chromatography-tandem mass spectrometry (LC-MS/MS) with a lower limit of quantification (LLOQ) of 2 pg/mL for both navafenterol and LAS191861, whilst for urine analyses an LC-MS/MS method was used with a LLOQ of 20 pg/mL for navafenterol.

Statistical Analysis

For safety analyses, AEs were coded using the Medical Dictionary for Regulatory Affairs version 18.1, and the number and percentage of patients with AEs were tabulated by body system and preferred term by treatment. Other safety variables were listed and summarised by treatment using descriptive statistics at each timepoint. Changes from day −1 were listed for clinical laboratory assessments, and changes from baseline were listed for blood pressure and 12-lead ECG parameters, where baseline was defined as the most recent pre-dose value on day 1 of each treatment period.

For PD analyses, baseline FEV_1, forced vital capacity and peak expiratory flow were the means of the values obtained at 45 and 15 min pre-dose on day 1 of each treatment period, ‘trough’ referred to the mean of the values obtained at 23 and 24 h post-dose, and ‘peak’

referred to the maximum value from the first 6 h post-dose. For the primary PD endpoint, an 'exploratory' statistical analysis was performed using three analysis of covariance models, which each assessed two dose levels (50 µg and 200 µg; 400 µg and 900 µg; and 1800 µg and 2100 µg) vs placebo. These were fitted separately and adjusted for treatment and baseline. The same model was used to assess the change from baseline in peak FEV_1 and normalised FEV_1 $AUC_{0–24}$. All statistical comparisons used 2-sided hypothesis tests, and descriptive statistics were presented by treatment for observed secondary PD variable values and changes from baseline.

PK parameters were summarised by dose group. Dose-proportionality analyses were performed on the PK population using a power model approach. Point estimates and 2-sided 95% confidence intervals were presented, and dose proportionality was defined if the slope did not significantly differ from 1.

E-TABLE 1. Safety Assessments

	Screening	Treatment Periods			Follow up
Assessment	Visit 1 (Day −28 to −2)	Visit 2 (Admission, Day −1)	Visit 3 (Day 1)	Visit 4 (Day 2)	Visit 5 (7 ±2 days after last dose)
Physical examination[a]	**X**		**X**	**X**	**X**
Vital signs					
Temperature[b]	**X**		**X**	**X**	**X**
Blood pressure[c]	**X**		**X**	**X**	**X**
Clinical laboratory tests[d]					
Chemistry	**X**	**X**		**X**	**X**
Haematology	**X**	**X**		**X**	**X**
Urinalysis	**X**	**X**		**X**	**X**
Coagulation	**X**				
Blood potassium and glucose[e]			**X**	**X**	
12-lead digital ECG[f]	**X**		**X**	**X**	**X**
Telemetry[g]		**X**	**X**	**X**	

ECG = electrocardiogram.

[a]A full examination was performed at screening and follow up, and a brief examination was performed pre-dose and before discharge at each treatment period.

[b]Determined at baseline and post-dose on day 1 (1, 6, 12, and 24 h) and day 2 (36 h).

[c]Determined in supine position at baseline ≤1 h prior to administration of study medication, and post-dose on day 1 (10 and 30 min, and 1, 2, 3, 4, 8, and 12 h) and day 2 (24 and 36 h).

[d]Chemistry, haematology, and urinalysis were performed post-dose on day 2 (24 h). Thyroid-stimulating hormone and thyroxine tests were performed at screening, at day −1, and at follow up.

[e]Determined at baseline ≤1 h prior to administration of study medication, and post-dose on day 1 (30 min and 1, 2, 4, and 12 h) and day 2 (24 h). Determined using i-STAT, with blood samples obtained in fasting conditions (≥ 4 h).

[f]Performed in triplicate; recorded at baseline ≤1 h prior to administration of study medication, and post-dose on day 1 (10 and 30 min, and 1, 2, 3, 4, 8, and 12 h) and day 2 (24 and 36 h).

[g]Telemetry with at least 2 lead real time display; recorded on day −1 (4–6 h continuous recording) and on day 1 until 24–36 h post-dose on day 2.

E-TABLE 2. Secondary Pharmacodynamic Endpoints (Per Protocol Population)

Parameter (L) (change from baseline)	Placebo (N = 12)	navafenterol 50 µg (N = 6)	navafenterol 200 µg (N = 6)	navafenterol 400 µg (N = 6)	navafenterol 900 µg (N = 6)	navafenterol 1800 µg (N = 6)	navafenterol 2100 µg (N = 5)
Trough FVC on day 2	−0.1263 (0.2903)	0.0633 (0.3405)	0.0117 (0.1536)	0.1833 (0.1753)	−0.0450 (0.3128)	0.1817 (0.1729)	0.2840 (0.4311)
Normalised FEV_1							
$AUC_{0–6}$	0.047 (0.204)	0.342 (0.291)	0.522 (0.104)	0.653 (0.129)	0.474 (0.174)	0.613 (0.248)	0.702 (0.156)
$AUC_{0–12}$	−0.071 (0.420)	0.225 (0.273)	0.490 (0.148)	0.562 (0.066)	0.416 (0.263)	0.581 (0.224)	0.673 (0.114)
$AUC_{12–24}$	−0.054 (0.241)[a]	−0.078 (0.371)[a]	0.254 (0.179)	0.278 (0.116)	0.225 (0.375)	0.379 (0.136)	0.494 (0.160)
$AUC_{0–24}$	−0.002 (0.217)	0.092 (0.306)	0.372 (0.147)	0.420 (0.075)	0.321 (0.318)	0.480 (0.176)	0.584 (0.123)
Peak FEV_1 on day 1	0.191 (0.108)	0.513 (0.295)	0.628 (0.091)	0.820 (0.219)	0.582 (0.128)	0.719 (0.271)	0.870 (0.145)

$AUC_{0–6}$ = area under the curve from 0 to 6 h; $AUC_{0–12}$ = area under the curve from 0 to 12 h; $AUC_{12–24}$ = area under the curve from 12 to 24 h; $AUC_{0–24}$ = area under the curve from 0 to 24 h; FEV_1 = forced expiratory volume in 1 second; FVC = forced vital capacity.

All data are mean (standard deviation).

[a]For placebo and navafenterol 50 µg, the total number of patients differed from the number of non−missing observations; there were 11 and 5, respectively.

E-TABLE 3. PK Parameters for navafenterol (PK Population)

Parameter	navafenterol 50 µg	navafenterol 200 µg	navafenterol 400 µg	navafenterol 900 µg	navafenterol 1800 µg	navafenterol 2100 µg
$AUC_{0-\infty}$ (pg.h/mL)						
n	5	6	6	6	6	5
Geometric mean	123.1	774.5	1671	3831	6941	7164
GCV%	75.48	32.64	28.54	28.72	22.23	34.10
AUC_{0-24} (pg.h/mL)						
n	6	6	6	6	6	5
Geometric mean	118.9	567.9	1265	3020	5587	5922
GCV%	60.19	29.74	32.44	25.71	25.40	34.32
C_{max} (pg/mL)						
n	6	6	6	6	6	5
Geometric mean	34.77	174.1	313.4	797.3	1351	1243
GCV%	60.77	43.74	46.56	20.99	20.32	24.33
t_{max} (h)						
n	6	6	6	6	6	5

Median	0.88	0.86	1.00	1.00	1.49	1.02
Min, max	0.52, 1.03	0.48, 1.00	0.75, 1.05	0.98, 1.02	0.73, 2.03	1.00, 2.00
$t_{½λz}$ (h)						
n	5	6	6	6	6	5
Arithmetic mean	9.439	23.10	20.81	18.86	19.29	15.96
SD	7.350	2.809	5.384	3.959	5.468	2.789
fe_{0-36h} (%)						
n	6	6	6	6	6	5
Arithmetic mean	0.1846	0.3789	0.2418	0.3459	0.2994	0.2477
SD	0.1101	0.1922	0.1223	0.1619	0.1314	0.09601
Renal clearance (L/h)						
n	6	6	6	6	6	5
Arithmetic mean	0.7209	1.102	0.6722	0.9413	0.9046	0.8021
SD	0.1020	0.2798	0.3267	0.4354	0.4143	0.2661

$AUC_{0-\infty}$ = area under the concentration-time curve from time zero extrapolated to infinity; AUC_{0-24} = area under the curve from 0 to 24 h; C_{max} = maximum plasma concentration; fe_{0-36h} = fraction of dose excreted unchanged into urine from 0 to 36 h; GCV% = geometric coefficient of variation; max = maximum; min = minimum;

n = number of non−missing observations; PK = pharmacokinetic; SD = standard deviation; $t_{½λz}$ = terminal elimination half−life; t_{max} = time to reach the maximum plasma concentration.

e-Figure 1 – *The chemical structure of navafenterol*

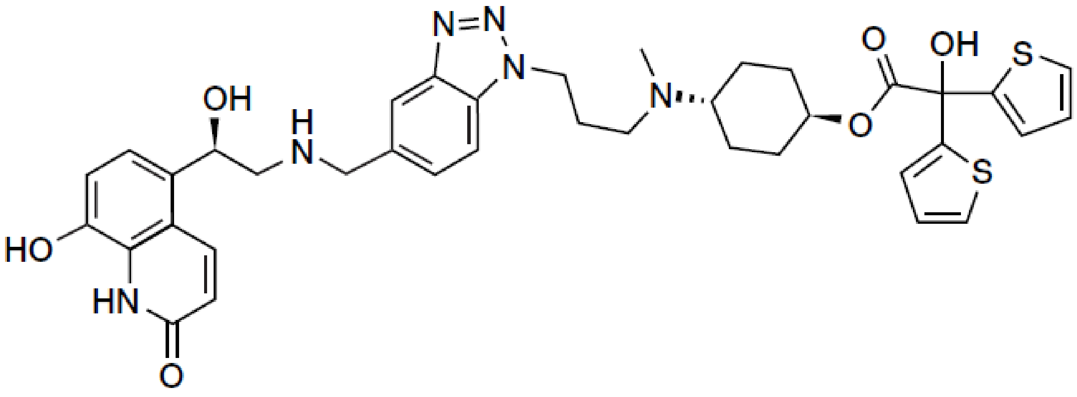

e-Figure 2 – *Mean (SD) change from baseline in (A) serum glucose and (B) serum potassium over 24 h post-dose (safety population). SD = standard deviation.*

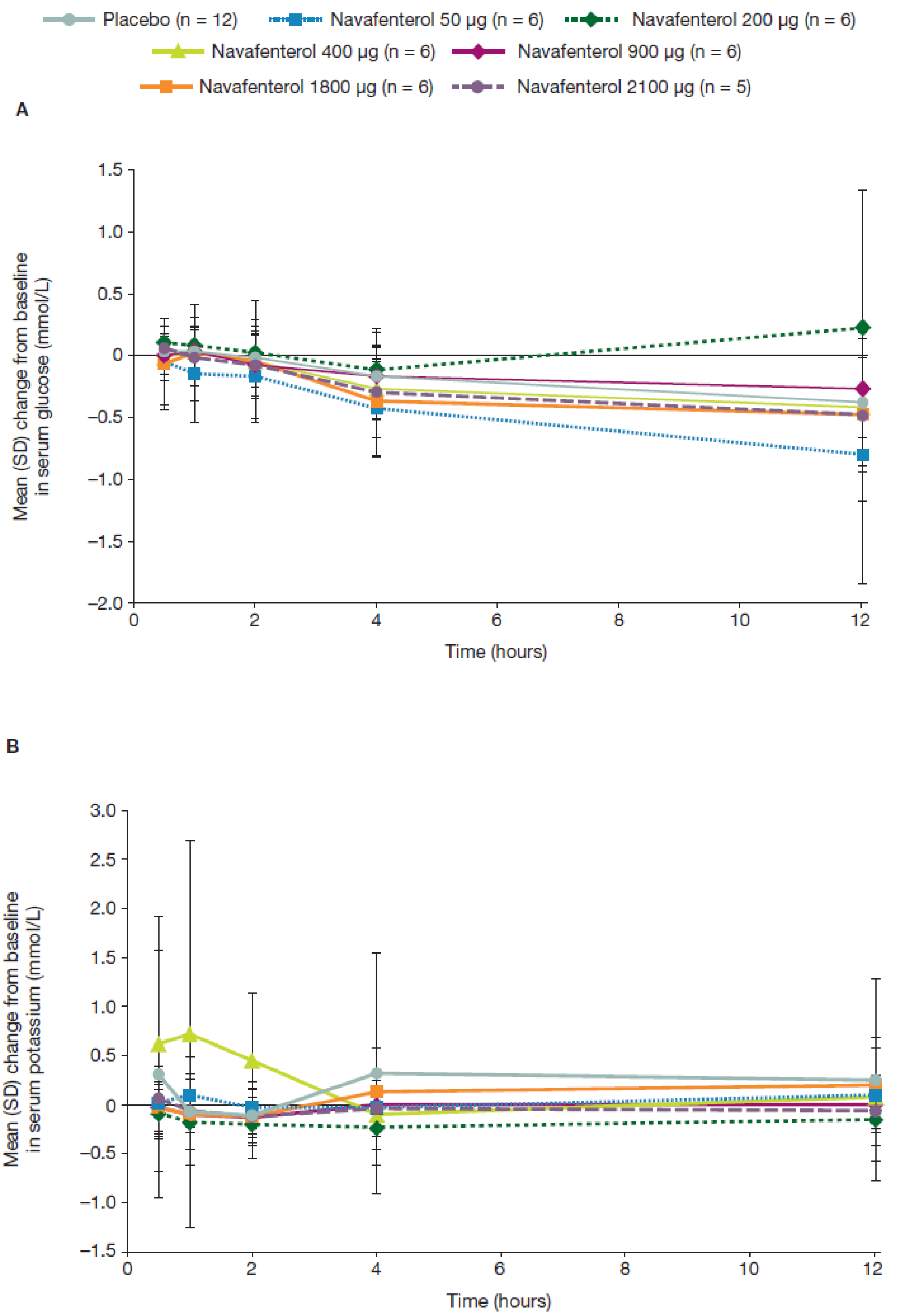

e-Figure 3 – *Mean (95% CI) change from baseline in* FEV_1 *at each timepoint over 36 h post-dose (per protocol population). The total number of patients differed from the number of non−missing observations for placebo (at 12 h and 14 h; both n = 11), navafenterol 50 µg (at 14 h; n = 5), and navafenterol 200 µg (at 5 min; n = 5). CI = confidence interval;* FEV_1 *= forced expiratory volume in 1 second.*

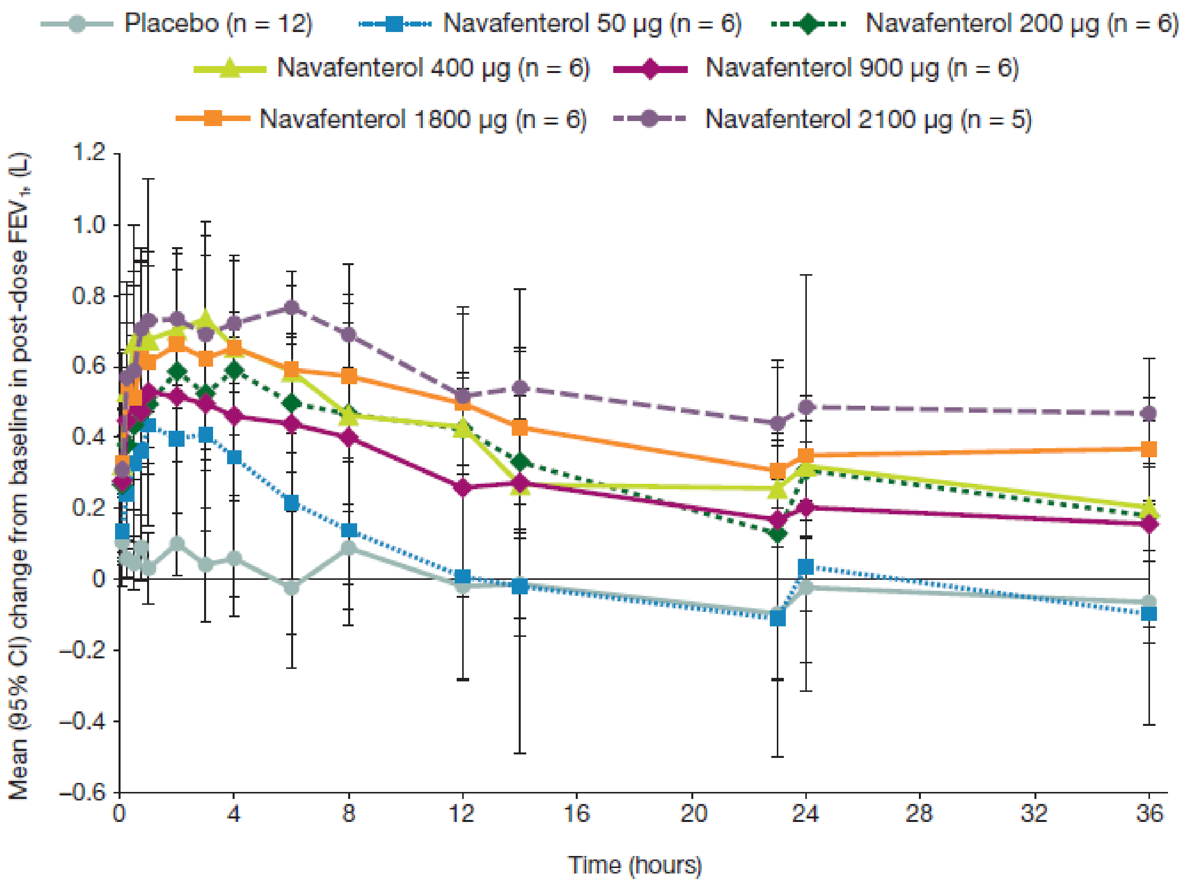

Supplement: Supplementary file 1 — Additional file 1 e-Appendix 1. Methods. E-Table 1. Safety Assessments. E-Table 2. Secondary Pharmacodynamic Endpoints. E-Table 3. PK Parameters for navafenterol. e-Figure 1. The chemical structure of navafenterol. e-Figure 2. Mean (SD) change from baseline in (A) serum glucose and (B) serum potassium over 24 h post-dose. e-Figure 3. Mean (95% CI) change from baseline in FEV1 at each timepoint over 36 h post-dose. [file 12931_2020_1470_MOESM1_ESM.pdf]
